# Transition between 2019 and 2025 sarcopenia definitions by the Asian Working Group for Sarcopenia in community-dwelling older adults

**DOI:** 10.1186/s12877-026-07849-1

**Published:** 2026-06-24

**Authors:** Nath Adulkasem, Ekasame Vanitcharoenkul, Aasis Unnanuntana, Pojchong Chotiyarnwong

**Affiliations:** Department of Orthopaedic Surgery, Faculty of Medicine, Siriraj Hospital, Mahidol University, 2 Wanglang Road, Bangkoknoi, Bangkok, 10700 Thailand

**Keywords:** Sarcopenia, Thailand, AWGS2025, Community-Dwelling, Transition

## Abstract

**Background:**

The Asian Working Group for Sarcopenia (AWGS) has revised its diagnostic criteria over time, most recently in 2025. The impact of these updates on sarcopenia classification in real-world Asian populations has not been fully established. This study compared the characteristics and diagnostic transitions of community-dwelling older adults between the 2019 and 2025 definitions.

**Methods:**

We conducted a secondary analysis of a nationwide epidemiological study of community-dwelling Thai adults aged ≥ 60 years. Muscle mass was measured using bioelectrical impedance analysis, handgrip strength with a digital dynamometer and physical performance using gait speed, five-times sit-to-stand and timed up-and-go tests. Sarcopenia was classified according to AWGS 2019 and 2025 definitions. Diagnostic transitions between the 2019 and 2025 criteria were visualized using a Sankey diagram. Characteristics and functional measures were then compared between concordant and reclassified groups.

**Results:**

From overall 2,456 subjects (Male: Female = 894: 1,562), sarcopenia prevalence increased following the AWGS definition update (18.1% in 2019 vs. 28.3% in 2025; *p* < 0.001). The AWGS 2025 definition identified more individuals with higher BMI and those aged < 65 years, while functional profiles remained comparable to those identified by the 2019 definition. For possible sarcopenia, prevalence decreased substantially under AWGS 2025 compared with AWGS 2019 (51.2% vs. 93.7%; *p* < 0.001). Diagnostic transition analysis showed that 14.9% of participants were newly classified as sarcopenic under AWGS 2025, while 4.7% were reclassified as non-sarcopenia. Characteristics and functional profiles of the reclassified sarcopenia group were similar to the concordant sarcopenia group, though long-term clinical outcomes remain to be established. Participants reclassified to non-sarcopenia showed no clear distinction from either concordant group, signaling potential diagnostic overlap at the margins.

**Conclusions:**

The AWGS 2025 criteria identify a broader population with sarcopenia, including younger individuals and those with higher BMI, while remaining largely consistent with the AWGS 2019 diagnostic definition. While these similarities suggest that the updated framework may enhance sensitivity to detect individuals with underlying vulnerabilities, further prospective studies are required to determine if these newly captured individuals experience true, long-term adverse clinical outcomes or if this expansion introduces a risk of diagnostic overcounting. The observed ambiguity in those reclassified as non-sarcopenic further highlights that while the updated framework may improve practical applicability, a clear line between high-risk phenotype and overdiagnosis remains difficult to draw using cross-sectional indices alone. The findings of this study are limited to individuals aged ≥ 60 years, and the application of the 2025 criteria to younger populations requires further investigation. Longitudinal studies are also needed to refine age-specific cut-offs and to clarify the clinical significance of individuals near diagnostic thresholds.

**Supplementary Information:**

The online version contains supplementary material available at https://doi.org/10.1186/s12877-026-07849-1.

## Introduction

Sarcopenia is an age-related condition characterized by the loss of skeletal muscle mass and deterioration of muscle function [1]. The term derives from *sarx* meaning flesh and *penia* meaning deficiency, literally referring to the loss of muscle mass [2]. Similar to other degenerative disorders, changes in both the quantity and quality of skeletal muscle help explain the age-related decline in locomotion, strength and functional capacity [1, 3]. However, defining clinically relevant sarcopenia is challenging. Quantifying muscle mass alone does not reliably reflect disease severity, and current approaches to assessing muscle quality lack consistency across studies [4, 5]. Consequently, diagnostic criteria continue to evolve to better capture both structural and functional decline and to guide clinical care [3, 6, 7].

Diverse human characteristics across the world including ethnicity, lifestyle and regional health disparities add further complexity to defining sarcopenia. In response, experts in Asia formed the Asian Working Group for Sarcopenia (AWGS) to develop criteria tailored to Asian populations. AWGS released its first consensus in 2014, aiming to provide guidance that reflects Asian body composition, strength measurements and performance patterns based on available evidence to promote sarcopenia research in Asian countries [3]. In 2019, AWGS updated its consensus with a stronger focus on primary care and community prevention [6]. The group introduced the concept of “possible sarcopenia,” defined by low muscle strength or poor physical performance without requiring muscle mass measurement. This approach was designed to support early detection in settings where diagnostic resources are limited and to promote timely preventive interventions. These two guidelines have formed the foundation of sarcopenia research in recent years and have influenced multiple clinical practice guidelines and their implementation [1, 7–9].

In 2025, AWGS released its most updated guideline to further enhance practicality across different clinical settings [7]. The guideline expands its scope from older adults to middle-aged adults, framing sarcopenia not only as a geriatric condition but also as a broader concept of muscle health and longevity. It also refines the definition and diagnostic algorithm to improve precision and to align with global initiatives such as the World Health Organization’s Integrated Care for Older People (ICOPE) framework and the Global Leadership Initiative in Sarcopenia (GLIS) [10, 11].

Despite the strong principles behind these guidelines, evaluating the population-level impact and clinical characteristics associated with the updated criteria is essential to determine their appropriateness and guide future refinement. This study aims to compare the prevalence and clinical characteristics of sarcopenia according to the updated AWGS criteria in a nationally representative sample of community-dwelling older adults. We characterize what the updated definition newly captures, describing the proportion and clinical profile of individuals who shift between sarcopenic and non-sarcopenic classifications under the revised criteria. This approach allows us to evaluate whether newly identified individuals share clinically meaningful characteristics with those consistently classified as sarcopenic, and to identify subgroups whose classification warrants further investigation.

## Methods

This study is a secondary analysis of a nationwide epidemiological study conducted in Thailand from 2021 to 2022. The original survey used a stratified multistage sampling method to ensure balanced representation from all six geographic regions and aimed to assess osteoporosis, sarcopenia and fall risk among community-dwelling older adults. Thai adults aged 60 years or older were eligible, while those with major health conditions that prevented physical performance testing were excluded. Demographic and clinical information was collected, including age, sex, weight, height, body mass index and comorbidities. Ethical approval for this study was granted by the Siriraj Institutional Review Board, which waived the requirement for additional informed consent given the secondary nature of the data analysis. All participants had previously provided written informed consent during the original study enrollment.

### Appendicular skeletal muscle mass (ASM) evaluation

ASM was assessed using a bioelectrical impedance analysis (BIA) device (RD-545; Tanita Corporation, Tokyo, Japan). All measurements were performed by trained investigators following standardized procedures. Participants fasted for at least four hours prior to assessment to minimize the effect of hydration status, and all measurements were conducted within a consistent timeframe to improve reliability [12, 13].

### Muscle strength

A Smedley-type digital handgrip dynamometer (TKK 5401; Takei, Tokyo, Japan) was used to measure grip strength. Participants completed the test while standing with their elbows fully extended and were instructed to squeeze with maximal effort for both hands [14]. The highest recorded value was used for analysis.

### Physical performance

This study assessed physical performance using gait speed, the five-times sit-to-stand test (FTSTS) and the timed up-and-go (TUG) test [15]. Gait speed was measured by recording the time participants took to walk six meters at their usual pace on an even surface [6]. For the five-times sit-to-stand test, we recorded the time required to complete five consecutive rises from an upright seated position in an armless chair with the arms crossed [16]. For the TUG test, participants were timed while standing up from a chair, walking three meters, turning around, walking back and sitting down again [17].

### Sarcopenia definition

Sarcopenia was classified according to the 2019 and 2025 criteria [6, 7]. Although the AWGS 2025 consensus expanded its scope to include middle-aged adults starting from 50 years old, our study population was restricted to individuals aged ≥ 60 years, reflecting the enrollment criteria of the original nationwide epidemiological survey. Applying the AWGS 2025 framework to this cohort remains highly clinically relevant, as it contains individuals spanning both distinct diagnostic tiers defined by the 2025 update: the newly established middle-aged subgroup (specifically those aged 60–64 years, evaluated using younger age-specific thresholds) and the older adult subgroup (aged ≥ 65 years). Evaluating this cohort allows us to capture the immediate real-world impact of transitioning between diagnostic tiers within a single aging sample. Under the AWGS 2019 definition, sarcopenia required low appendicular skeletal muscle mass (ASM), defined as height-adjusted ASM < 7.0 kg/m² in men and < 5.7 kg/m² in women, in combination with either low muscle strength (handgrip strength < 28 kg in men and < 18 kg in women) or impaired physical performance (gait speed < 1.0 m/s or 5-time chair stand test ≥ 12 s) [6]. The AWGS 2025 criteria introduced age-specific cut-offs and alternative ASM indices. For individuals aged ≥ 65 years, low ASM was defined as height-adjusted ASM < 7.0 kg/m² in men and < 5.7 kg/m² in women, or BMI-adjusted ASM < 0.83 in men and < 0.57 in women. For those aged 50–64 years, the cut-offs were < 7.6 kg/m² in men and < 5.7 kg/m² in women for height-adjusted ASM, or < 0.90 in men and < 0.63 in women for BMI-adjusted ASM. Muscle strength thresholds were < 28 kg in men and < 18 kg in women for ≥ 65 years, and < 34 kg in men and < 20 kg in women for those aged 50–64 years [7]. In the 2025 definition, sarcopenia could be diagnosed based on low muscle strength alone, while physical performance was reserved for outcome assessment in accordance with the Global Leadership Initiative in Sarcopenia (GLIS) framework [10]. Full definitions are presented in Supplementary Table 1.

### Health-related quality of life and fall risk assessment

Health-related quality of life was assessed using the EQ-5D visual analogue scale (EQ VAS). Participants rated their overall health on a scale from 0 to 100, where 0 represents the worst imaginable health state and 100 represents the best imaginable health state [18].

Fall risk was assessed using a self-rated fall risk questionnaire (FRQ), a validated screening tool for identifying individuals at risk of falls. The questionnaire consists of items related to balance, gait, prior falls, and functional status, with a total score ranging from 0 to 14. A score of ≥ 4 was used to define increased fall risk, based on established cut-off values from the original validation study [19].

### Statistical analysis

All analyses were performed using Stata Statistical Software, release 16 (StataCorp LLC, College Station, TX, USA). Continuous variables are presented as mean ± standard deviation and categorical variables as counts and percentages. Group comparisons were conducted using t-tests, Mann–Whitney U tests, McNemar’s test, or Fisher’s exact tests as appropriate. A p-value < 0.05 was considered statistically significant.

The prevalence of sarcopenia based on each AWGS definition was presented across different age groups. Participant characteristics and functional measures for “sarcopenia” and “possible sarcopenia” were described using both the AWGS 2019 and AWGS 2025 diagnostic criteria stratified by gender. To examine diagnostic shifts between the previous and updated guidelines, we constructed a Sankey diagram illustrating transitions between AWGS 2019 and AWGS 2025. Participants were grouped into four categories: *concordant sarcopenia*, defined as individuals diagnosed with sarcopenia by both AWGS 2019 and AWGS 2025; *concordant non-sarcopenia*, defined as those classified as non-sarcopenia by both versions; *reclassified sarcopenia*, referring to individuals initially labelled non-sarcopenic under AWGS 2019 but newly diagnosed as sarcopenic under AWGS 2025; and *reclassified non-sarcopenia*, referring to those diagnosed with sarcopenia under AWGS 2019 but no longer meeting diagnostic criteria under AWGS 2025. We then compared characteristics and functional outcomes across these four groups to evaluate the discriminative performance of the older and updated criteria. These comparisons were based on prespecified contrasts designed to address specific clinical questions regarding reclassification under the updated criteria, rather than exhaustive pairwise comparisons across all group combinations. Formal correction for multiple comparisons was therefore not applied.

## Results

**Fig. 1:**
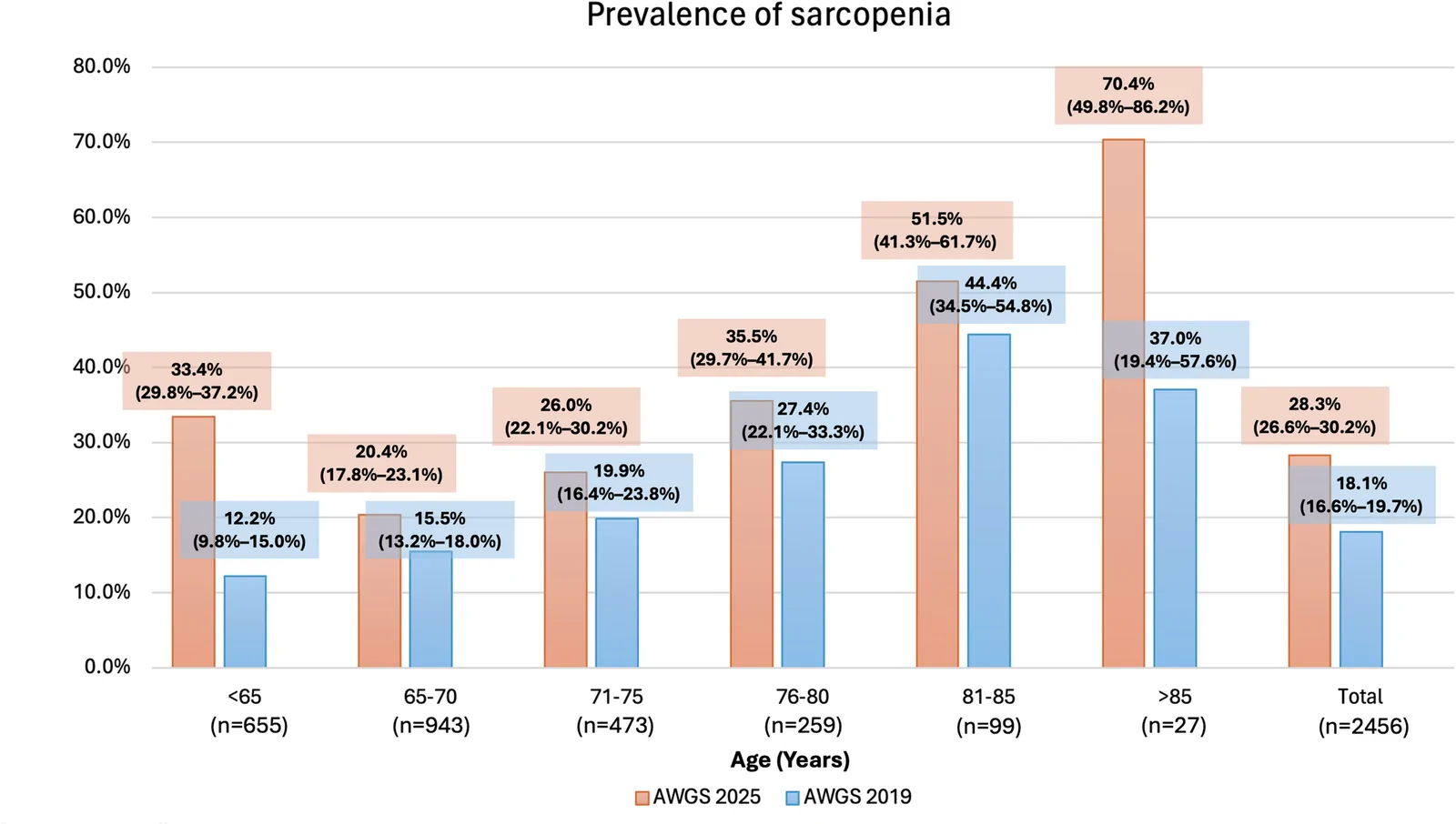
Age-stratified sarcopenia prevalence according to AWGS 2019 and AWGS 2025 criteria with 95% confidence intervals

A total of 2456 participants were included in the analysis. The prevalence of sarcopenia defined by AWGS 2025 was 28.3%, markedly higher than that identified using the AWGS 2019 definition (18.1%) (*p* < 0.001) (Table 1). When stratified by sex, men demonstrated consistently higher prevalence than women across both the AWGS 2019 and AWGS 2025 criteria, with an incremental increase observed with the 2025 update (Table 2). The AWGS 2025 definition also identified individuals who were younger and had higher BMI compared with those classified by the AWGS 2019 definition. Functional measures were otherwise comparable across both criteria within each sex. When examined across age groups, the AWGS 2025 criteria yielded higher prevalence estimates in every age category, with a notably higher prevalence among participants younger than 65 years (33.4%) (Fig. 1).

**Table 1:** Characteristics and function of sarcopenia among AWGS 2025, and 2019 definition

Characteristics	AWGS 2025 *n* = 696/2456 (28.3%)	AWGS 2019 *n* = 445/2456 (18.1%)
Age (years)	69.8 ± 7.3	71.3 ± 7.0
Sex, n (%)		
Male	326 (46.8%)	237 (53.3%)
Female	370 (53.2%)	208 (46.7%)
Weight (kg)	55.8 ± 12.0	48.9 ± 8.6
Height (cm)	154.5 ± 8.6	157.8 ± 8.1
BMI^a^ (kg/m^2^)	23.4 ± 5.1	19.6 ± 2.8
CCI^b^ (median (IQR))	3 (2, 4)	3 (2, 4)
Osteoporosis, n (%)	245 (35.2%)	211 (47.4%)
Handgrip strength (kg)	18.6 ± 5.8	20.3 ± 6.6
ASM^c^ (kg)	15.8 ± 3.4	14.5 ± 2.9
ASM/h^2^ (kg/m^2^)	6.6 ± 1.2	5.8 ± 0.7
ASM/BMI (m^2^)	0.69 ± 0.16	0.75 ± 0.16
Gait speed (m/s)	0.86 ± 0.20	0.87 ± 0.21
FTSTS^d^ test (s)	17.0 ± 5.2	16.7 ± 5.1
FTSTS test > 12 s	608 (87.4%)	395 (88.8%)
TUG^e^ test (s)	12.5 ± 4.0	12.3 ± 4.0
TUG > 12 s	341 (49.1%)	202 (45.5%)
EQ-VAS^f^	78.7 ± 62.9	78.9 ± 46.9
FRQ^g^	3.4 ± 3.2	3.0 ± 3.1
High fall risk (FRQ ≥ 4)	273 (39.3%)	148 (33.3%)

^a﻿^BMI, Body Mass Index, ^b^CCI, Charlson Comorbidities Index, ^c^ASM, Appendicular Muscle Mass, ^d^FTSTS, Five-Times-Sit-To-Stand test, ^e^TUG, Time-Up-and-Go test, ^f^EQ-VAS, EuroQol Visual Analogue Scale, ^g^FRQ, self-rated Falls Risk Questionnaire

**Table 2:** Characteristics and function of sarcopenia among AWGS 2025, and 2019 definition

Characteristics	Male (*n* = 894)		Female (*n* = 1562)	
	AWGS 2025 *n* = 326 (36.5%)	AWGS 2019 *n* = 237 (26.5%)	AWGS 2025 *n* = 370 (23.7%)	AWGS 2019 *n* = 208 (13.3%)
Age (years)	70.5 ± 7.3	71.3 ± 7.0	69.3 ± 7.2	71.3 ± 7.0
Weight (kg)	57.1 ± 11.1	51.7 ± 7.5	54.7 ± 12.8	45.8 ± 8.7
Height (cm)	160.8 ± 6.8	162.5 ± 7.0	149.0 ± 5.9	152.4 ± 5.5
BMI^a^ (kg/m^2^)	22.1 ± 4.1	19.5 ± 2.3	24.6 ± 5.6	19.6 ± 3.3
CCI^b^, median (IQR)	3 (2, 4)	3 (2, 4)	3 (2, 4)	3 (2, 4)
Osteoporosis, n (%)	72 (22.1%)	68 (28.7%)	173 (46.8%)	143 (68.8%)
Handgrip strength (kg)	22.8 ± 5.3	23.9 ± 6.0	14.9 ± 2.9	16.2 ± 4.5
ASM^c^ (kg)	18.0 ± 3.1	16.6 ± 2.2	13.7 ± 2.3	12.2 ± 1.4
ASM/h^2^ (kg/m^2^)	7.0 ± 1.2	6.3 ± 0.6	6.2 ± 1.0	5.2 ± 0.4
ASM/BMI (m^2^)	0.8 ± 0.1	0.9 ± 0.1	0.6 ± 0.1	0.6 ± 0.1
Gait speed (m/s)	0.9 ± 0.2	0.9 ± 0.2	0.8 ± 0.2	0.8 ± 0.2
FTSTS^d^ test (s)	15.4 ± 3.9	15.0 ± 3.4	18.4 ± 5.8	18.7 ± 6.1
FTSTS test > 12 s	271 (83.1%)	204 (86.1%)	337 (91.1%)	191 (91.8%)
TUG^e^ test (s)	11.3 ± 3.0	11.1 ± 2.9	13.7 ± 4.5	13.6 ± 4.6
TUG > 12 s	112 (34.4%)	75 (31.7%)	229 (62.2%)	127 (61.4%)
EQ-VAS^f^, median (IQR)	80 (70, 90)	80 (70, 90)	70 (60, 85)	80 (60, 90)
FRQ^g^, median (IQR)	1 (0, 3)	1 (0, 3)	4 (1, 7)	3 (1, 7)
High fall risk (FRQ ≥ 4)	73 (22.5%)	50 (21.1%)	200 (54.2%)	98 (47.1%)

^a﻿^BMI, Body Mass Index, ^b^CCI, Charlson Comorbidities Index, ^c^ASM, Appendicular Muscle Mass, ^d^FTSTS, Five-Times-Sit-To-Stand test, ^e^TUG, Time-Up-and-Go test, ^f^EQ-VAS, EuroQol Visual Analogue Scale, ^g^FRQ, self-rated Falls Risk Questionnaire

For possible sarcopenia, the prevalence was substantially lower under AWGS 2025 compared with AWGS 2019 (51.2% vs. 93.7%, *p* < 0.001) (Supplementary Table 2). This reduction reflects the narrower 2025 definition, which relies solely on handgrip strength (Supplementary Table 1), and the pattern was consistent in both sexes (Supplementary Table 3). Participant characteristics and functional measures were comparable between the two definitions, suggesting that both criteria identify a similar group of individuals who may benefit from early intervention.

**Fig. 2:**
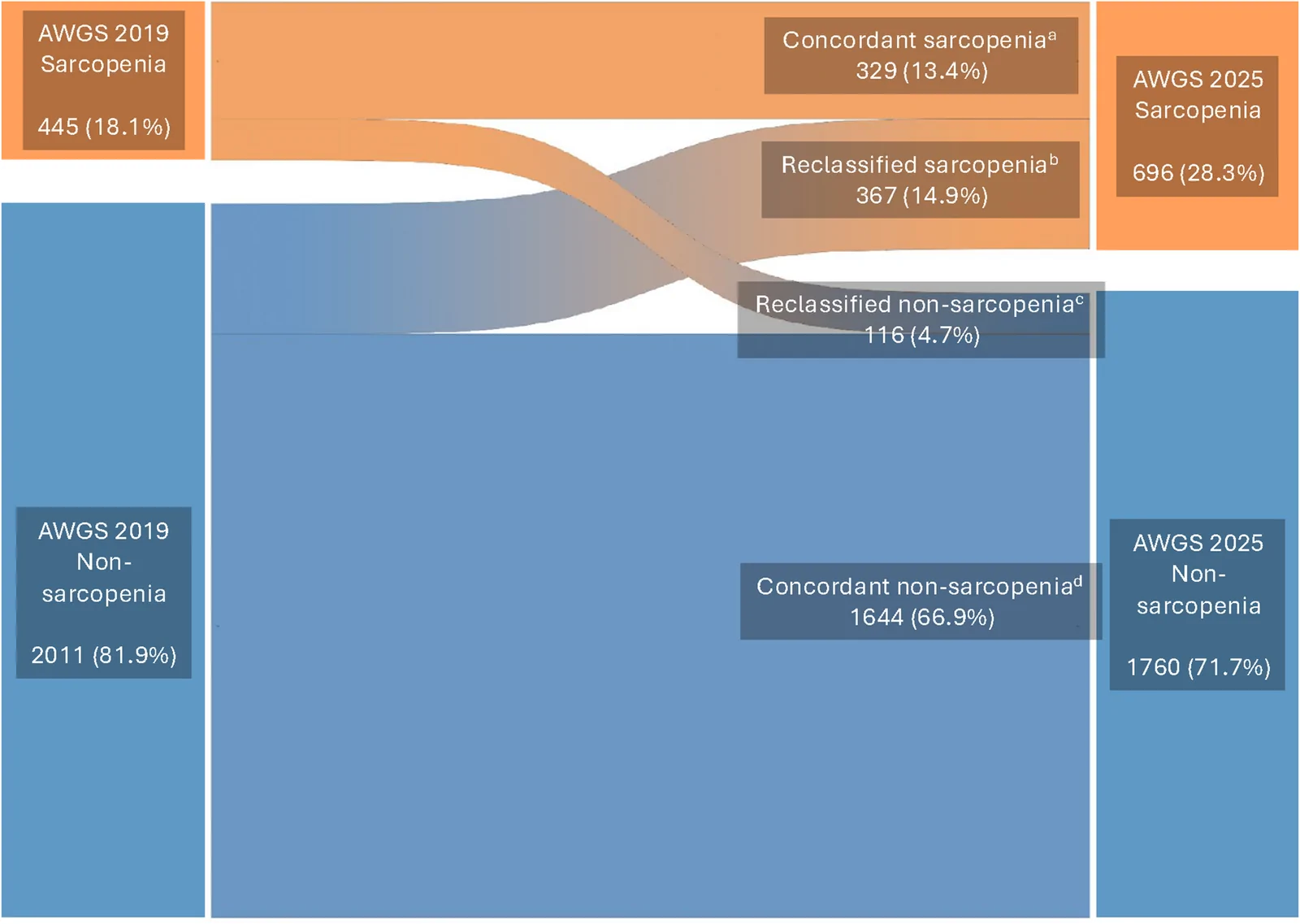
Diagram illustrating diagnostic transitions between AWGS 2019 and AWGS 2025 criteria. Among 2,456 participants, 445 (18.1%) were classified as sarcopenic and 2,011 (81.9%) as non-sarcopenic under AWGS 2019, while 696 (28.3%) and 1,760 (71.7%) were classified as sarcopenic and non-sarcopenic, respectively, under AWGS 2025. Of these, 329 (13.4%) were concordantly classified as sarcopenic by both criteria, and 1,644 (66.9%) were concordantly classified as non-sarcopenic. Reclassification occurred in 367 (14.9%) participants who were newly classified as sarcopenic under AWGS 2025 and 116 (4.7%) who were reclassified as non-sarcopenic

When examining diagnostic shifts between the AWGS 2019 and 2025 definitions, the Sankey diagram showed that 367 participants (14.9%) were reclassified to sarcopenia (*reclassified sarcopenia group*) and 116 participants (4.7%) were reclassified to non-sarcopenia (*reclassified non-sarcopenia group*) (Fig. 2). The remaining participants were classified as *concordant sarcopenia* (329 participants, 13.4%) or *concordant non-sarcopenia* (1,644 participants, 66.9%). The overall percent agreement between AWGS 2019 and AWGS 2025 classifications was 80.3%.

We compared the characteristics and functional measures of the reclassified groups with those of the concordant groups to highlight similarities and differences across classifications. The *reclassified sarcopenia group* had poorer physical performance and quality of life and higher fall risk than the *concordant non-sarcopenia group* (Table 3). When compared with the *concordant sarcopenia group*, this reclassified group showed comparable physical performance and quality of life but demonstrated a higher fall risk. In contrast, the reclassified non-sarcopenia group did not show meaningful differences in overall characteristics, including functional measures, when compared with either concordant group (Table 4). This lack of distinction was also evident when the two reclassified groups were compared directly (Supplementary Table 4). As expected, the *concordant sarcopenia* and *concordant non-sarcopenia groups* differed markedly in physical performance, whereas fall risk and quality of life remained similar (Supplementary Table 5).

**Table 3:** Characteristic and functional outcomes of newly reclassified sarcopenia cases under AWGS 2025 vs. concordant non-sarcopenic cases (AWGS 2019 and 2025)

Characteristics	Reclassified sarcopenic patients*	Concordant Sarcopenic patients**		Concordant non-sarcopenic patients***	
	*n* = 367	*n* = 329	*p*-value^†^	*n* = 1644	*p*-value^‡^
Gait speed (m/s)	0.87 ± 0.20	0.86 ± 0.2	0.626	0.93 ± 0.19	< 0.001
FTSTS^a^ test (s)	17.1 ± 5.1	16.8 ± 5.3	0.400	16.0 ± 4.6	< 0.001
FTSTS test > 12 s	321 (87.5%)	287 (87.2%)	1.000	1384 (84.2%)	0.127
TUG^b^ test (s)	12.6 ± 3.9	12.4 ± 4.2	0.549	11.3 ± 3.5	< 0.001
TUG > 12 s	189 (51.5%)	152 (46.2%)	0.149	567 (34.5%)	< 0.001
EQ-VAS^c^	80 (60, 85)	80 (60, 90)	0.183	80 (70, 90)	0.002
FRQ^d^	3 (1, 6)	2 (1, 5)	0.004	2 (0, 5)	< 0.001
High fall risk (FRQ ≥ 4)	166 (45.2%)	107 (32.5%)	0.001	570 (34.7%)	< 0.001

^a﻿^FTSTS, Five-Times-Sit-To-Stand test, ^b^TUG, Time-Up-and-Go test, ^c^EQ-VAS, EuroQol Visual Analogue Scale, ^d^FRQ, self-rated Falls Risk Questionnaire

^*^Participants who were classified as non-sarcopenic by AWGS 2019 but sarcopenic by AWGS 2025

^**^Participants classified as sarcopenic by both AWGS 2019 and AWGS 2025

^***^Participants classified as non-sarcopenic by both AWGS 2019 and AWGS 2025

^†^*p*-values represent comparisons between reclassified sarcopenic patients and concordant sarcopenic patients

^‡^*p*-values represent comparisons between reclassified sarcopenic patients and concordant non-sarcopenic patients

**Table 4:** Characteristics and functional outcomes of newly reclassified non-sarcopenia cases under AWGS 2025 vs. concordant non-sarcopenia cases (AWGS 2019 and 2025)

Characteristics	Reclassified Non-sarcopenic patients*	Concordant Sarcopenic patients**		Concordant non-sarcopenic patients***	
	*n* = 116	*n* = 329	*p*-value^†^	*n* = 1644	*p*-value^‡^
Gait speed (m/s)	0.90 ± 0.20	0.86 ± 0.21	0.103	0.93 ± 0.19	0.107
FTSTS^a^ test (s)	16.5 ± 4.5	16.8 ± 5.3	0.545	16.0 ± 4.6	0.331
FTSTS test > 12 s	108 (93.1%)	287 (87.2%)	0.009	1384 (84.2%)	0.007
TUG^b^ test (s)	11.8 ± 3.4	12.4 ± 4.2	0.123	11.3 ± 3.5	0.174
TUG > 12 s	50 (43.1%)	152 (46.2%)	0.664	567 (34.5%)	0.069
EQ-VAS^c^	80 (70, 95)	80 (60, 90)	0.009	80 (70, 90)	0.014
FRQ^d^	2 (0.5, 5)	2 (1, 5)	0.960	2 (0, 5)	0.801
High fall risk (FRQ ≥ 4)	41(35.3%)	107 (32.5%)	0.647	570 (34.7%)	0.920

^a﻿^FTSTS, Five-Times-Sit-To-Stand test, ^b^TUG, Time-Up-and-Go test, ^c^EQ-VAS, EuroQol Visual Analogue Scale, ^d^FRQ, self-rated Falls Risk Questionnaire

^*^Participants who were classified as sarcopenic by AWGS 2019 but non-sarcopenic by AWGS 2025

^**^Participants classified as sarcopenic by both AWGS 2019 and AWGS 2025

^***^Participants classified as non-sarcopenic by both AWGS 2019 and AWGS 2025

^†^*p*-values represent comparisons between reclassified non-sarcopenic patients and concordant sarcopenic patients

^‡^*p*-values represent comparisons between reclassified non-sarcopenic patients and concordant non-sarcopenic patients

## Discussion

This study compared the two most recent iterations of the AWGS diagnostic criteria (AWGS 2019 and AWGS 2025) and demonstrated a progressive increase in sarcopenia prevalence in the updated criteria, indicating that newer definitions capture a broader segment of the population. The AWGS 2025 criteria identified more cases in both sexes, including individuals with higher BMI and those younger than 65 years. Despite the removal of physical performance from the diagnostic algorithm, these individuals exhibited strength, functional performance, quality of life and fall risk profiles similar to those diagnosed using the AWGS 2019 definition. The reclassification analysis further showed that AWGS 2025 identified an additional 14.9% of individuals whose clinical and functional profiles were broadly comparable to those with concordant sarcopenia. While this suggests that the updated criteria are sensitive to individuals with worse functional profiles compared to non-sarcopenic peers, it is premature to definitively rule out diagnostic overcounting; because our data are limited to a single time point, whether these newly captured cases represent clinically meaningful disease trajectories or an artificial inflation of prevalence remains an open question. It is worth acknowledging that grip strength, gait speed, and FTSTS form part of the diagnostic criteria themselves, and differences on these measures partly reflect the distinct threshold values applied by each definition. Nevertheless, the consistency observed across criterion-independent outcomes, including quality of life and fall risk scores, provides additional support for the clinical relevance of the updated criteria independent of their diagnostic construction.

The substantial increase in diagnoses under AWGS 2025 may reflect two key changes in the updated criteria. First, the criteria were expanded to include adults aged 50–64 years with newly derived age-specific cut-offs, a population not specifically targeted by AWGS 2019, which contributed a notable number of additional cases in the younger age group (Supplementary Table 6). Second, AWGS 2025 allows either height-adjusted or BMI-adjusted ASM to satisfy the muscle mass criterion, unlike AWGS 2019 which relied solely on height-adjusted ASM. To explore the contribution of this change, we performed a cross-tabulation within the reclassified sarcopenia group (Supplementary Table 7), which showed that a substantially higher proportion met the low muscle mass criterion via BMI-adjusted ASM but would not have qualified under height-adjusted ASM alone, suggesting that the introduction of BMI-adjusted ASM was an additional driver of new case identification. Previous literature supports this approach, showing that BMI-standardized ASM reduces the impact of body composition variability and is more strongly associated with muscle weakness, fall risk, and other adverse outcomes [7, 20, 21].

Separating physical performance evaluation from the diagnostic component did not diminish the ability of the updated criteria to detect sarcopenia. Individuals identified by the 2025 definition exhibited characteristics comparable to those classified by the 2019 version, indicating consistent case recognition across guideline versions. This approach is consistent with the GLIS recommendation that physical performance is more appropriate for outcome assessment rather than diagnostic classification [10]. Notably, removal of physical performance from the diagnostic algorithm also explains the 116 participants (4.7%) reclassified as non-sarcopenic under AWGS 2025, as individuals previously diagnosed based on poor physical performance alone no longer meet the updated criteria. Despite this, the comparable functional profiles observed across both sarcopenic groups suggest that this reclassification reflects a deliberate refinement of diagnostic focus toward muscle quantity and strength rather than a loss of clinically meaningful case identification. The revised guideline therefore provides several advantages, including improved feasibility in routine practice, fewer limitations related to performance testing, and earlier identification of individuals who may benefit from intervention.

Despite the higher sarcopenia prevalence, younger participants consistently demonstrated better physical function, lower fall risk, and lower comorbidity burden. Further examination showed that this discrepancy was primarily attributable to a substantially higher proportion of younger participants meeting the low BMI-adjusted ASM criterion, whereas differences in height-adjusted ASM and grip strength were less pronounced (Supplementary Table 6). These findings suggest that the elevated sarcopenia prevalence in adults aged < 65 years is largely driven by the BMI-adjusted ASM threshold introduced in AWGS 2025 rather than a true increase in sarcopenia burden. The age-specific cut-off values applied to individuals aged 50–64 years may create an abrupt classification boundary that is inconsistent with the gradual age-related decline in muscle mass and physical function observed in normative aging trajectories [15, 22]. Consequently, the current cut-off values may increase the likelihood of classifying otherwise healthy community-dwelling individuals as sarcopenic, and their clinical applicability warrants further validation. Nevertheless, these findings should also be interpreted in the context of our study population, because the cohort was restricted to individuals aged ≥ 60 years. As a result, individuals aged 50–59 years, who represent an important component of the newly defined AWGS 2025 age category, were not included. Consequently, the prevalence estimates reported for the < 65-year group may not be representative of the broader 50–64-year population and may overestimate the prevalence that would be observed in a complete middle-aged cohort. Further studies involving the full 50–64-year age range are needed to validate the performance of these cut-off values. Alternative approaches, such as population-based percentile thresholds or T-score–based definitions, may also merit investigation, as they may better reflect the continuous nature of age-related changes in muscle mass across the lifespan.

The reclassification analysis supports the clinical consistency of the AWGS 2025 definition. Participants reclassified to sarcopenia demonstrated lower muscle strength, reduced muscle mass, and poorer functional performance than the concordant non-sarcopenia group, with characteristics more closely resembling those with concordant sarcopenia. This shared profile indicates that the newly identified individuals exhibit a risk cluster that aligns with a sarcopenic phenotype. However, distinguishing between a highly sensitive, clinically meaningful diagnostic threshold and overdiagnosis is impossible without longitudinal outcome data. For instance, some of these individuals may possess subclinical variations in body composition that trigger the new BMI-adjusted threshold without ever progressing to actual clinical impairment. However, their younger age, higher BMI, and greater absolute muscle mass under height-adjusted indices may indicate a distinct phenotype, potentially reflecting early sarcopenic obesity that is better captured by BMI-adjusted assessment. Although some differences were modest, the overall pattern supports the clinical relevance of this reclassification. Confirming whether this represents a distinct phenotype would require longitudinal outcome data beyond the scope of the current study.

In contrast, participants reclassified as non-sarcopenic under AWGS 2025 appeared to represent a borderline group whose optimal classification remains uncertain. These individuals previously met AWGS 2019 criteria through low muscle mass and impaired physical performance despite preserved grip strength, a diagnostic pathway no longer recognized in AWGS 2025. This pattern may reflect an early stage of sarcopenic progression in which lower limb function declines before grip strength, consistent with evidence demonstrating that quadriceps and lower limb muscle strength decline at a greater rate than handgrip strength with advancing age [23]. Their reclassification therefore likely reflects a change in diagnostic criteria rather than a true improvement in muscle health. We suggest that individuals in this subgroup undergo periodic reassessment of muscle strength and mass, as a previous sarcopenia diagnosis may still indicate underlying vulnerability despite no longer meeting the updated criteria. Prospective longitudinal studies are needed to clarify the natural history of these individuals and determine whether they remain at increased risk of functional decline, falls, or progression to overt sarcopenia.

This study has several strengths, including the use of a large, nationally representative cohort, standardized assessments and direct comparison across both versions of the AWGS criteria. Nonetheless, several limitations should be considered. First, our cohort included only participants aged ≥ 60 years, meaning the 50–59 age bracket introduced by the AWGS 2025 guidelines was entirely unrepresented. While this allowed us to investigate the application of the newly proposed 50–64 age thresholds on individuals transitioning into older adulthood (60–64 years), it limits our ability to evaluate the full scope of the AWGS 2025 epidemiological shift in true middle-aged adults. This structural truncation likely overinflated the sarcopenia prevalence reported for our youngest subgroup. Second, muscle mass was assessed using dual-frequency bioelectrical impedance analysis (BIA) rather than the recommended multi-frequency BIA, which may reduce accuracy in body composition estimation, particularly in individuals with extreme body composition or altered hydration status. However, dual-frequency devices have shown comparable accuracy for estimating appendicular skeletal muscle mass (ASM) and have been validated against dual-energy X-ray absorptiometry (DXA) in similar populations [12]. In addition, we minimized measurement variability by standardizing all procedures, including participant preparation and measurement conditions, in accordance with recommended protocols. Third, although comparisons were based on prespecified group contrasts rather than exhaustive pairwise testing, the number of variables compared across groups increases the risk of type I error in the absence of formal multiple comparison correction. Results should therefore be interpreted with appropriate caution. Fourth, the cross-sectional design precludes assessment of longitudinal outcomes, limiting evaluation of future clinical outcomes and long-term implications. Finally, the findings may not be generalizable beyond community-dwelling older adults without major health conditions. Future studies should incorporate longitudinal outcome follow-up, refine age-specific cut-off values and further investigate the clinical significance of borderline classifications.

## Conclusions

The AWGS 2025 criteria identify a broader population with sarcopenia, including younger individuals and those with higher BMI, while remaining largely consistent with the AWGS 2019 diagnostic definition. However, some individuals reclassified as non-sarcopenic did not differ clearly from either concordant group, suggesting potential uncertainty in classification at the lower-risk boundary. While this updated framework potentially enhances the early identification of individuals exhibiting adverse muscle health features, the sharp increase in prevalence underscores the critical need to investigate potential overdiagnosis. Nonetheless, the observed ambiguity in those reclassified as non-sarcopenic indicates that further refinement of the criteria is needed. The findings of this study are limited to individuals aged ≥ 60 years, and the application of the 2025 criteria to younger populations requires further investigation. Longitudinal studies are urgently needed to track the actual incidence of adverse events in these newly reclassified cohorts, refine age-specific cut-offs, and clarify the clinical significance of individuals near diagnostic thresholds.

## Supplementary Information

Supplementary Material 1.

## Data Availability

The datasets generated and/or analyzed during the current study are not publicly available due to institutional and ethical restrictions but are available from the corresponding author upon reasonable request.

## References

[1] Vanitcharoenkul E, Unnanuntana A, Chotiyarnwong P, Laohaprasitiporn P, Adulkasem N, Asavamongkolkul A, Chandhanayingyong C. Sarcopenia in Thai community-dwelling older adults: a national, cross-sectional, epidemiological study of prevalence and risk factors. BMC Public Health. 2024;24(1):311. 38281041 10.1186/s12889-024-17804-7 PMC10821311

[2] Sanchez-Tocino ML, Cigarrán S, Ureña P, González-Casaus ML, Mas-Fontao S, Gracia-Iguacel C, Ortíz A, Gonzalez-Parra E. Definition and evolution of the concept of sarcopenia. Nefrología. 2024;44(3):323–30. 38945744 10.1016/j.nefroe.2023.08.007

[3] Chen LK, Liu LK, Woo J, Assantachai P, Auyeung TW, Bahyah KS, Chou MY, Chen LY, Hsu PS, Krairit O, et al. Sarcopenia in Asia: consensus report of the Asian Working Group for Sarcopenia. J Am Med Dir Assoc. 2014;15(2):95–101. 24461239 10.1016/j.jamda.2013.11.025

[4] Jiménez-Lupión D, Chirosa-Ríos I, Lizama-Pérez R, Chirosa-Ríos L, Jerez-Mayorga D. Assessment of muscle function deterioration in aging populations: Insights from the load-velocity relationship during the loaded sit-to-stand test. Exp Gerontol. 2025;206:112767. 40316224 10.1016/j.exger.2025.112767

[5] Beaudart C, Rolland Y, Cruz-Jentoft AJ, Bauer JM, Sieber C, Cooper C, Al-Daghri N, Araujo de Carvalho I, Bautmans I, Bernabei R, et al. Assessment of Muscle Function and Physical Performance in Daily Clinical Practice. Calcif Tissue Int. 2019;105(1):1–14. 30972475 10.1007/s00223-019-00545-w

[6] Chen LK, Woo J, Assantachai P, Auyeung TW, Chou MY, Iijima K, Jang HC, Kang L, Kim M, Kim S, et al. Asian Working Group for Sarcopenia: 2019 Consensus Update on Sarcopenia Diagnosis and Treatment. J Am Med Dir Assoc. 2020;21(3):300–e307302. 32033882 10.1016/j.jamda.2019.12.012

[7] Chen LK, Hsiao FY, Akishita M, Assantachai P, Lee WJ, Lim WS, et al. A focus shift from sarcopenia to muscle health in the Asian Working Group for Sarcopenia 2025 Consensus Update. Nat Aging. 2025;5(11):2164–2175. 10.1038/s43587-025-01004-y. 41188603

[8] Asavamongkolkul A, Adulkasem N, Chotiyarnwong P, Vanitcharoenkul E, Chandhanayingyong C, Laohaprasitiporn P, Soparat K, Unnanuntana A. Prevalence of osteoporosis, sarcopenia, and high falls risk in healthy community-dwelling Thai older adults: a nationwide cross-sectional study. JBMR Plus. 2024;8(2):ziad020. 38505534 10.1093/jbmrpl/ziad020 PMC10945715

[9] Adulkasem N, Vanitcharoenkul E, Chotiyarnwong P, Asavamongkolkul A, Unnanuntana A. Evaluation of the Diagnosis Accuracy of the AWGS 2019 Criteria for Possible Sarcopenia in Thai Community-Dwelling Older Adults. Clin Interv Aging. 2025;20:425–33. 40230404 10.2147/CIA.S513657 PMC11994469

[10] Kirk B, Cawthon PM, Arai H, Ávila-Funes JA, Barazzoni R, Bhasin S, et al. The Conceptual Definition of Sarcopenia: Delphi Consensus from the Global Leadership Initiative in Sarcopenia (GLIS). Age Ageing. 2024;53(3):afae052. 10.1093/ageing/afae052. PMC10960072 38520141

[11] WHO Guidelines Approved by the Guidelines Review Committee. In: Integrated Care for Older People: Guidelines on Community-Level Interventions to Manage Declines in Intrinsic Capacity. Geneva: World Health Organization Copyright © World Health Organization; 2017. 29608259

[12] Anusitviwat C, Vanitcharoenkul E, Chotiyarnwong P, Unnanuntana A. Dual-Frequency Bioelectrical Impedance Analysis is Accurate and Reliable to Determine Lean Muscle Mass in The Elderly. J Clin Densitom. 2023;26(1):90–6. 36567160 10.1016/j.jocd.2022.12.006

[13] Kyle UG, Bosaeus I, De Lorenzo AD, Deurenberg P, Elia M, Manuel Gómez J, Lilienthal Heitmann B, Kent-Smith L, Melchior J-C, Pirlich M, et al. Bioelectrical impedance analysis—part II: utilization in clinical practice. Clin Nutr. 2004;23(6):1430–53. 15556267 10.1016/j.clnu.2004.09.012

[14] Roberts HC, Denison HJ, Martin HJ, Patel HP, Syddall H, Cooper C, Sayer AA. A review of the measurement of grip strength in clinical and epidemiological studies: towards a standardised approach. Age Ageing. 2011;40(4):423–9. 21624928 10.1093/ageing/afr051

[15] Chandhanayingyong C, Adulkasem N, Asavamongkolkul A, Chotiyarnwong P, Vanitcharoenkul E, Laohaprasitiporn P, Soparat K, Unnanuntana A. Establishing Normative Values for Performance-Based Tests in Older Thai Adults: A Nationwide Cross-Sectional Study. Arch Phys Med Rehabil. 2024;105(6):1133–41. 38367833 10.1016/j.apmr.2024.01.023

[16] Albalwi AA, Alharbi AA. Optimal procedure and characteristics in using five times sit to stand test among older adults: A systematic review. Med (Baltim). 2023;102(26):e34160. 10.1097/MD.0000000000034160 PMC10313281 37390277

[17] Podsiadlo D, Richardson S. The timed Up & Go: a test of basic functional mobility for frail elderly persons. J Am Geriatr Soc. 1991;39(2):142–8. 1991946 10.1111/j.1532-5415.1991.tb01616.x

[18] Chuang LH, Kind P, Kohlmann T, Feng YS. Exploring the origin and conceptual framework of the EQ VAS. Qual Life Res. 2025;34(8):2163–73. 40285933 10.1007/s11136-025-03947-6 PMC12274267

[19] Kitcharanant N, Vanitcharoenkul E, Unnanuntana A. Validity and reliability of the self-rated fall risk questionnaire in older adults with osteoporosis. BMC Musculoskelet Disord. 2020;21(1):757. 33208120 10.1186/s12891-020-03788-z PMC7677850

[20] Kinoshita K, Satake S, Matsui Y, Arai H. Association between Sarcopenia and Fall Risk According to the Muscle Mass Adjustment Method in Japanese Older Outpatients. J Nutr health aging. 2021;25(6):762–6. 34179931 10.1007/s12603-021-1620-8 PMC12876577

[21] Tang T-C, Hwang A-C, Liu L-K, Lee W-J, Chen L-Y, Wu Y-H, Huang C-Y, Hung C-H, Wang C-J, Lin M-H, et al. FNIH-defined Sarcopenia Predicts Adverse Outcomes Among Community-Dwelling Older People in Taiwan: Results From I-Lan Longitudinal Aging Study. Journals Gerontology: Ser A. 2018;73(6):828–34. 10.1093/gerona/glx148 28977377

[22] Wang YC, Bohannon RW, Li X, Sindhu B, Kapellusch J. Hand-Grip Strength: Normative Reference Values and Equations for Individuals 18 to 85 Years of Age Residing in the United States. J Orthop Sports Phys Ther. 2018;48(9):685–93. 29792107 10.2519/jospt.2018.7851

[23] Samuel D, Wilson K, Martin HJ, Allen R, Sayer AA, Stokes M. Age-associated changes in hand grip and quadriceps muscle strength ratios in healthy adults. Aging Clin Exp Res. 2012;24(3):245–50. 23114550 10.1007/BF03325252

