# Supplementary material for: Transition between 2019 and 2025 sarcopenia definitions by the Asian Working Group for Sarcopenia in community-dwelling older adults
Source: BMC Geriatr. 2026 Jun 24;26:1147. doi: 10.1186/s12877-026-07849-1 (PMC13556062; doi:10.1186/s12877-026-07849-1)
Supplement: Supplementary file 1 [file 12877_2026_7849_MOESM1_ESM.docx]

**Supplementary table**

**Supplementary Table 1.** Definition of sarcopenia according to Asian Working Group for Sarcopenia (AWGS) 2025 and 2019

| **Diagnosis criteria** | **AWGS 2025** | | **AWGS 2019** |
| --- | --- | --- | --- |
|  | Age ≥64 years | Age 50-64 years |  |
| **Appendicular skeletal muscle mass (ASM)** | | | |
| Bioelectrical impedance analysis |  |  |  |
| Height-adjusted (kg/m^-2^) | M:<7.0, F: <5.7 | M:<7.6, F: <5.7 | M:<7.0, F: <5.7 |
| BMI-adjusted | M:<0.83, F: <0.57 | M:<0.90, F: <0.63 |  |
| *and* | | | |
| **Muscle function evaluation** | | | |
| 1. Muscle strength |  | | |
| Hand grip strength (kg) | M: <28, F <18 | M: <34, F <20 | M: <28, F <18 |
| *or* | | | |
| 2. Physical performance |  | |  |
| Gait speed (m/s) |  |  | <1.0 |
| 5-time chair stand test (s) |  |  | ≥12 |

| **Supplementary table 2.** Characteristics and function of **possible sarcopenia** between AWGS 2025 and AWGS 2019 definition | | |
| --- | --- | --- |
|  | **AWGS 2025**  n=1257/2456 (51.2%) | **AWGS 2019**  n=2302/2456 (93.7%) |
| Age (years) | 69.8±6.9 | 69.2±6.2 |
| Sex, n (%) |  |  |
| Male | 534 (42.5%) | 803 (34.9%) |
| Female | 723 (57.5%) | 1499 (65.1%) |
| Weight (kg) | 56.8±11.5 | 58.9±11.7 |
| Height (cm) | 155.3±8.3 | 155.8±8.1 |
| BMI^1^ (kg/m^2^) | 23.5±4.3 | 24.2±4.4 |
| CCI^2^ (median (IQR)) | 3 (2, 4) | 3 (2, 4) |
| Osteoporosis, n (%) | 394 (31.3%) | 651 (28.3%) |
| Handgrip strength (kg) | 18.4±5.7 | 21.4±6.7 |
| ASM^3^ (kg) | 16.9±4.1 | 17.2±4.0 |
| ASM/h^2^ (kg/m^2^) | 6.9±1.3 | 7.0±1.2 |
| ASM/BMI (m^2^) | 0.69±0.16 | 0.75±0.16 |
| Gait speed (m/s) | 0.87±0.20 | 0.89±0.19 |
| FTSTS^4^ test (s) | 16.9±5.1 | 16.7±4.6 |
| FTSTS test >12 s | 1104 (87.8%) | 2100 (91.2%) |
| TUG^5^ test (s) | 12.4±4.0 | 11.9±3.7 |
| TUG >12 s | 590 (47.1%) | 953 (41.6%) |
| EQ-VAS^6^ | 79.3±65.9 | 79.0±56.8 |
| FRQ^7^ | 3.4±3.2 | 3.1±3.0 |
| High fall risk (FRQ≥4) | 515 (41.1%) | 867 (37.7%) |
| Sarcopenia diagnosis* | 696 (55.4%) | 445 (19.3%) |
| ^1^BMI, Body Mass Index; ^2^CCI, Charlson Comorbidities Index; ^3^ASM, Appendicular Muscle Mass; ^4^FTSTS, Five-Times-Sit-To-Stand test; ^5^TUG, Time-Up-and-Go test; ^6^EQ-VAS, EuroQol Visual Analogue Scale; ^7^FRQ, self-rated Falls Risk Questionnaire  *Sarcopenia diagnosis based on the corresponding AWGS version | | |

| **Supplementary Table 3.** Characteristics and function of **possible sarcopenia** between AWGS 2025 and AWGS 2019 definition by gender | | | | |
| --- | --- | --- | --- | --- |
| Demographic | **Male (n=894)** | | **Female (n=1562)** | |
|  | **AWGS 2025**  n=534 (59.7%) | **AWGS 2019**  n=803 (89.8%) | **AWGS 2025**  n=723 (46.3%) | **AWGS 2019**  n=1499 (96.0%) |
| Age (years) | 70.0±7.0 | 69.9±6.4 | 69.7±6.8 | 68.8±6.0 |
| Weight (kg) | 59.8±11.3 | 62.0±11.7 | 54.6±11.0 | 57.2±11.3 |
| Height (cm) | 161.8±6.2 | 162.9±6.2 | 150.5±6.1 | 152.0±6.2 |
| BMI^1^ (kg/m^2^) | 22.8±3.9 | 23.3±4.0 | 24.1±4.5 | 24.7±4.5 |
| CCI^2^ (median (IQR)) | 3 (2, 4) | 3 (2, 4) | 3 (2, 4) | 3 (2, 4) |
| Osteoporosis, n (%) | 94 (17.6%) | 115 (14.3%) | 300 (41.5%) | 536 (35.8%) |
| Handgrip strength (kg) | 23.0±5.4 | 26.5±7.0 | 15.0±2.9 | 18.7±4.7 |
| ASM^3^ (kg) | 19.9±3.9 | 20.7±3.9 | 14.6±2.2 | 15.3±2.4 |
| ASM/h^2^ (kg/m^2^) | 7.6±1.4 | 7.8±1.3 | 6.4±0.9 | 6.6±0.9 |
| ASM/BMI (m^2^) | 0.9±0.1 | 0.9±0.1 | 0.6±0.1 | 0.6±0.1 |
| Gait speed (m/s) | 0.9±0.2 | 0.9±0.2 | 0.8±0.2 | 0.9±0.2 |
| FTSTS^4^ test (s) | 15.3±3.8 | 15.2±3.6 | 18.2±5.6 | 17.6±4.9 |
| FTSTS test >12 s | 445 (83.3%) | 697 (86.8%) | 659 (91.2%) | 1403 (93.6%) |
| TUG^5^ test (s) | 11.1±2.9 | 10.9±2.7 | 13.3±4.5 | 12.4±4.1 |
| TUG >12 s | 176 (33.0%) | 231 (28.8%) | 414 (57.7%) | 722 (48.2%) |
| EQ-VAS^6^ | 80 (70, 90) | 80 (70, 90) | 80 (60, 80) | 80 (60, 90) |
| FRQ^7^ | 1 (0, 3) | 1 (0, 3) | 4 (1, 7) | 3 (1, 6) |
| High fall risk (FRQ≥4) | 124 (23.2%) | 171 (21.3%) | 391 (54.1%) | 696 (46.4%) |
| Sarcopenia diagnosis* | 326 (61.1%) | 237 (29.5%) | 370 (51.2%) | 208 (13.9%) |
| ^1^BMI, Body Mass Index; ^2^CCI, Charlson Comorbidities Index; ^3^ASM, Appendicular Muscle Mass; ^4^FTSTS, Five-Times-Sit-To-Stand test; ^5^TUG, Time-Up-and-Go test; ^6^EQ-VAS, EuroQol Visual Analogue Scale; ^7^FRQ, self-rated Falls Risk Questionnaire  *Sarcopenia diagnosis based on the corresponding AWGS version | | | | |

| **Supplementary table 4.** Characteristics and functional outcomes of newly reclassified sarcopenia cases vs. newly reclassified non-sarcopenia cases under AWGS 2025 | | | |  |
| --- | --- | --- | --- | --- |
| Characteristics | **Reclassified**  **sarcopenic patients***  n=367 (76.0%) | **Reclassified**  **non-sarcopenic patients****  n=116 (24.0%) | **p-value** |  |
| Gait speed (m/s) | 0.87±0.20 | 0.90±0.20 | 0.171 |  |
| FTSTS^1^ test (s) | 17.1±5.1 | 16.5±4.5 | 0.206 |  |
| FTSTS test >12 s | 321 (87.5%) | 108 (93.1%) | 0.127 |  |
| TUG^2^ test (s) | 12.6±3.9 | 11.8±3.4 | 0.174 |  |
| TUG >12 s | 189 (51.8%) | 50 (43.5%) | 0.135 |  |
| EQ-VAS^3^ | 80 (60, 85) | 80 (70, 95) | <0.001 |  |
| FRQ^4^ | 3 (1, 6) | 2 (0.5, 5) | <0.001 |  |
| High fall risk (FRQ≥4) | 166 (45.5%) | 41 (35.3%) | 0.067 |  |
| *Participants who were classified as sarcopenic by AWGS 2019 but non-sarcopenic by AWGS 2025 | | | |  |
| **Participants who were classified as non- sarcopenic by AWGS 2019 but sarcopenic by AWGS 2025 | | | |  |
| ^1^FTSTS, Five-Times-Sit-To-Stand test; ^2^TUG, Time-Up-and-Go test; ^3^EQ-VAS, EuroQol Visual Analogue Scale; ^4^FRQ, self-rated Falls Risk Questionnaire | | | | |

| **Supplementary table 5.** Characteristics and functional outcomes of concordant sarcopenia cases vs. concordant non-sarcopenia cases (AWGS 2019 and 2025) | | | |  |
| --- | --- | --- | --- | --- |
| Characteristics | **Concordant**  **sarcopenic patients***  n=329 (16.7%) | **Concordant**  **non-sarcopenic patients****  n=1644 (83.3%) | **p-value** |  |
| Gait speed (m/s) | 0.86±0.21 | 0.93±0.19 | <0.001 |  |
| FTSTS^1^ test (s) | 16.8±5.3 | 16.0±4.6 | 0.007 |  |
| FTSTS test >12 s | 287 (87.2%) | 1384 (84.2%) | 0.180 |  |
| TUG^2^ test (s) | 12.4±4.2 | 11.3±3.5 | <0.001 |  |
| TUG >12 s | 152 (46.2%) | 567 (34.6%) | <0.001 |  |
| EQ-VAS^3^ | 80 (60, 90) | 80 (70, 90) | 0.254 |  |
| FRQ^4^ | 2 (1, 5) | 2 (0, 5) | 0.686 |  |
| High fall risk (FRQ≥4) | 107 (32.5%) | 570 (34.7%) | 0.484 |  |
| * Participants classified as sarcopenic by both AWGS 2019 and AWGS 2025 | | | |  |
| ** Participants classified as non-sarcopenic by both AWGS 2019 and AWGS 2025 | | | |  |
| ^1^FTSTS, Five-Times-Sit-To-Stand test; ^2^TUG, Time-Up-and-Go test; ^3^EQ-VAS, EuroQol Visual Analogue Scale; ^4^FRQ, self-rated Falls Risk Questionnaire | | | | |

| **Supplementary Table 6**. Comparison of characteristics between populations aged <65 and ≥65 years | | | |
| --- | --- | --- | --- |
|  | Age<65 n=655 | Age≥65 n=1801 | p-value |
| Sarcopenia (AWGS2025) ^*^ | 33.4% (29.8%-37.2%) | 26.5% (24.5%-28.6%) | 0.001 |
| Sarcopenia (AWGS2019) ^*^ | 12.2% (9.8%-15.0%) | 20.3% (18.4%-22.2%) | <0.001 |
| Sex, n (%) |  |  |  |
| Male | 213 (33.9%) | 681 (37.8%) | 0.018 |
| Female | 442 (67.5%) | 1120 (62.2%) |  |
| Weight (kg) | 60.7±11.6 | 58.3±11.5 | <0.001 |
| Height (cm) | 156.6±8.1 | 155.9±8.1 | 0.076 |
| BMI^1^ (kg/m^2^) | 24.8±4.5 | 24.0±4.2 | <0.001 |
| CCI^2^ (median (IQR)) | 2 (2, 3) | 3 (2, 4) | <0.001 |
| ASM^3^ (kg) | 17.8±4.0 | 17.1±4.0 | <0.001 |
| Low BMI-adjusted ASM | 293 (44.7%) | 530 (29.4%) | <0.001 |
| Low Height-adjusted ASM | 112 (17.1%) | 375 (20.8%) | 0.045 |
| Low hand grip strength | 355 (54.2%) | 902 (50.1%) | 0.075 |
| Gait speed (m/s) | 1.0±0.2 | 0.9±0.2 | <0.001 |
| FTSTS^4^ test (s) | 15.6±4.5 | 16.6±4.8 | <0.001 |
| TUG^5^ test (s) | 10.9±3.2 | 12.0±3.9 | <0.001 |
| EQ-VAS^6^ | 76.9±15.2 | 80.2±63.7 | 0.193 |
| FRQ^7^ | 2.5±2.7 | 3.2±3.0 | <0.001 |

^*^Percentage with 95% Confidence Interval

^1^BMI, Body Mass Index; ^2^CCI, Charlson Comorbidities Index; ^3^ASM, Appendicular Muscle Mass; ^4^FTSTS, Five-Times-Sit-To-Stand test; ^5^TUG, Time-Up-and-Go test; ^6^EQ-VAS, EuroQol Visual Analogue Scale; ^7^FRQ, self-rated Falls Risk Questionnaire

| **Supplementary Table 7.** Overlap between height-adjusted and BMI-adjusted ASM criteria for low muscle mass under AWGS 2025 | | | |
| --- | --- | --- | --- |
|  | Sarcopenic by height-adjusted ASM | Not sarcopenic by height-adjusted ASM | Total |
| Sarcopenic by BMI-adjusted ASM | 161 (6.6%) | 340 (13.8%) | 501 (20.4%) |
| Not sarcopenic by BMI-adjusted ASM | 195 (7.9%) | 1,760 (71.7%) | 1,955 (79.6%) |
| Total | 356 (14.5%) | 2,100 (85.5%) | 2,456 (100%) |
